# Ventricular Morphology and Outcomes in Fontan Circulation without Hypoplastic Left Heart Syndrome: A Single-Center's Experience

**DOI:** 10.31083/j.rcm2506193

**Published:** 2024-05-27

**Authors:** Han Wang, Jianrui Ma, Linjiang Han, Tong Tan, Wen Xie, Miao Tian, Zichao Tujia, Ying Li, Xiang Liu, Xiaobing Liu, Haiyun Yuan, Jimei Chen

**Affiliations:** ^1^Department of Cardiovascular Surgery, Guangdong Cardiovascular Institute, Guangdong Provincial People's Hospital, Guangdong Academy of Medical Sciences, 510080 Guangzhou, Guangdong, China; ^2^Guangdong Provincial Key Laboratory of South China Structural Heart Disease, 510080 Guangzhou, Guangdong, China; ^3^Shantou University Medical College, 515041 Shantou, Guangdong, China; ^4^Department of Cardiovascular Surgery Center, Beijing Anzhen Hospital, Capital Medical University, Beijing Institute of Heart, Lung and Blood Vascular Diseases, 100029 Beijing, China

**Keywords:** Fontan, single ventricle, ventricular morphology, right ventricle, death or transplantation, Fontan failure

## Abstract

**Background:**

The impact of dominant ventricular morphology on Fontan patient outcomes remain controversial. This study evaluates long-term results of right ventricle (RV) dominance versus left ventricle (LV) dominance in Fontan circulation without hypoplastic left heart syndrome (HLHS).

**Methods:**

We retrospectively examined 323 Fontan operations from our center. To minimize pre- and intra-Fontan heterogeneity, 42 dominant RV patients were matched with 42 dominant LV patients using propensity score matching, allowing for a comparative analysis of outcomes between groups.

**Results:**

The mean follow-up was 8.0 ± 4.6 years for matched RV dominant and 6.5 ± 4.7 years for matched LV dominant group (*p* > 0.05), showing no significant difference. The cumulative incidence of moderate or greater atrioventricular valve regurgitation was also comparable between the two groups (*p* > 0.05). Similarly, 10-year freedom from death or transplantation following the Fontan operation was 84% ± 7% in the matched dominant RV group, similar to 81% ± 7% in the matched dominant LV group (*p* > 0.05). The 10-year freedom from Fontan failure was 78% ± 8% in the matched dominant RV group, also similar to 75% ± 8% in the matched dominant LV group (*p >*0.05). Multivariate analysis did not identify RV dominance as a risk factor for Fontan failure (*p* > 0.05).

**Conclusions:**

In the pre- and intra-Fontan context, RV dominance demonstrated similar and comparable long-term outcomes compared to LV dominance in non-HLHS Fontan circulation.

## 1. Introduction

Single ventricle (SV) deficits are rare congenital heart defects characterized by either one severely underdeveloped ventricle, or the absence of the ventricular septum, resulting in a broad range of cardiac structural abnormalities [1, 2]. This life-threatening congenital heart defect necessitates prompt intervention, commonly through the Fontan operation [2, 3, 4]. This Fontan procedure, which may be executed in either one or multiple stages as a total cavopulmonary connection, provides palliation and treatment, achieving satisfactory long-term survival for SV patients [3, 4]. However, it carries risks of both cardiac and non-cardiac complications that could lead to Fontan failure, primarily due to decreased cardiac output and persistent elevated systemic venous pressure from the absence of a sub-pulmonary ventricle [5, 6]. Consequently, extensive research is underway to identify risk factors and improve Fontan techniques, aiming to enhance patient outcomes.

Morphological variations SV deficits can be categorized as left, right, or indeterminate. It is hypothesized that Fontan patients with a dominant right ventricle (RV) experience worse outcomes compared to those with a dominant left ventricle (LV), potentially due to differences in anatomy, embryological origin, and affiliated atrioventricular valves [7, 8]. Nevertheless, the impact of RV dominance on Fontan procedure outcomes continues to be a controversial issue [9, 10, 11], likely influenced by the significant heterogeneity observed in Fontan patients.

Despite evidence from previous studies suggesting that RV dominance in Fontan circulation, particularly among patients with hypoplastic left heart syndrome (HLHS), leads to poorer outcomes compared to those with LV-dominance [12, 13], the reasons for this discrepancy are multifaceted. It’s speculated that the theoretical disadvantages of RV dominance are compounded by factors such as increased aortic stiffness and higher systemic afterload, outcomes often associated with Norwood surgery, further disadvantaging RV dominant systems [14, 15]. The connection between ventricular dominance and patient outcomes in non-HLHS Fontan circulation, however, remains unclear and contentious.

This ambiguity highlights the need for a nuanced understanding of how ventricular dominance influences long-term health and recovery in Fontan patients. Considering this complexity, we developed a propensity score matching (PSM) system aimed at reducing patient heterogeneity. This method enabled us to conduct a direct comparison of long-term outcomes between RV and LV dominance in Fontan patients, specifically excluding those with HLHS.

## 2. Methods

### 2.1 Population and Study Design

**Fig. 1.**
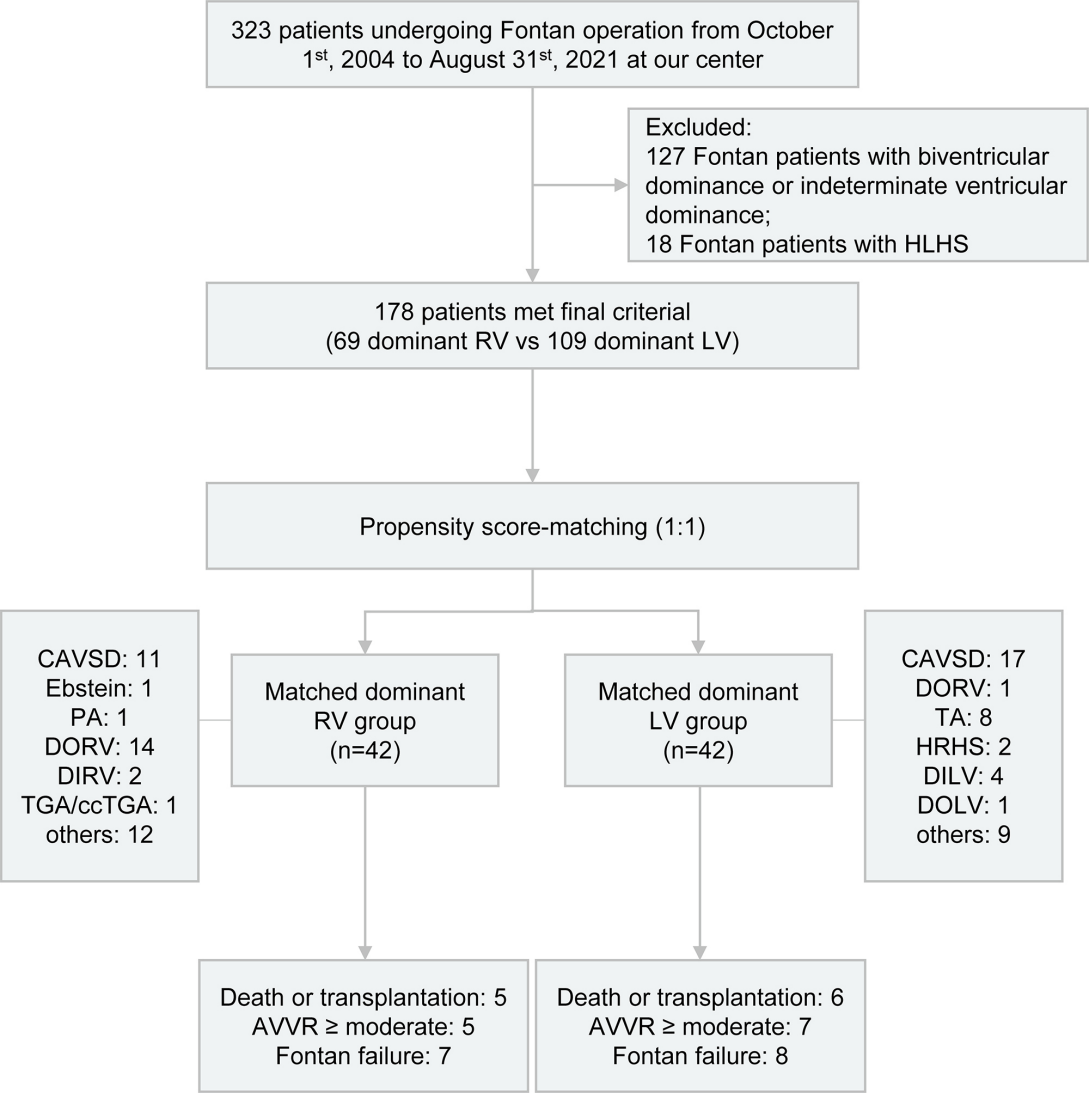
**Flowchart depicting the study design and outcomes for Fontan patients categorized by either dominant RV or LV.** HLHS, hypoplastic left heart syndrome; RV, right ventricle; LV, left ventricle; CAVSD, complete atrioventricular septal defect; PA, pulmonary atresia; DORV, double outlet right ventricle; DIRV, double inlet right ventricle; TGA, transposition of the great arteries; ccTGA, congenitally corrected transposition of the great arteries; TA, tricuspid atresia; HRHS, hypoplastic right heart syndrome; DILV, double inlet left ventricle; DOLV, double outlet left ventricle; AVVR, atrioventricular valve regurgitation.

This was a single-center retrospective study of 323 patients who underwent Fontan operation from October 1st, 2004 to August 31st, 2021 at our center, and was performed as illustrated in Fig. 1. 127 Fontan patients with biventricular dominance or indeterminate ventricular dominance and 18 Fontan patients with HLHS were excluded. Eventually, a total of 178 patients with RV or LV dominance were enrolled in this study.

### 2.2 Data Collection and Follow-Up

Ventricular morphological information and other baseline characteristics as well as perioperative and postoperative data were all reviewed and collected from the medical records of each patient. All medical records of enrolled patients were collected and extracted. The outpatient follow-up appointments were scheduled to be performed at 3, 6, and 12 months following the Fontan operation and annually thereafter. The follow-up echocardiograms were available in 190 of 196 patients (96.9%). Atrioventricular valve regurgitation (AVVR) assessed by echocardiogram was recorded as Grade 0 (none or trivial), Grade 1 (mild), Grade 2 (moderate), and Grade 3 (severe).

### 2.3 Endpoints and Definition

The primary outcomes were death or transplantation and Fontan failure during the follow-up. The secondary outcome was AVVR ≥ moderate during the follow-up. The composite endpoint of Fontan failure was defined as death or transplantation, Fontan conversion, Fontan takedown, protein-losing enteropathy, plastic bronchitis, and New York Heart Association functional class ≥III during the follow-up. In-hospital reintervention was defined as any unplanned operation after the Fontan operation occurring in hospital or within 30 days. In-hospital mortality was defined as any post-Fontan death occurring in hospital or within 30 days.

### 2.4 Statistical Analysis

All data analysis were performed using SPSS software (version 26, IBM SPSS statistics, Chicago, IL, USA) and R software (version 4.1.3, R Foundation for Statistical Computing, Vienna, Austria). Categorical variables were reported as numbers with percentages and compared between the two groups using the chi-square test. Continuous variables were reported as mean with standard deviation if normally distributed or median with interquartile range if not normally distributed. The student’s *t*-test and Mann-Whitney U test were utilized for comparisons between groups. Given the large heterogeneity in Fontan patients, PSM was used to minimize the potential selection. Propensity scores were calculated by logistic regression with variables (male sex, age at Fontan operation, weight at Fontan operation, Fontan type, Fontan fenestration, atrioventricular valve [AVV] morphology, atrial isomerism, dextrocardia, anomalous pulmonary vein connection, AVVR ≥ moderate, AVV operation before or at Fontan). The RV-dominant patients were matched with LV-dominant patients in a 1:1 ratio using the nearest neighbor method and a caliper of 0.2, yielding 55 pairs in total. The balance of baseline characteristics was assessed using standardized mean difference. Time-to-events including death or transplantation and Fontan failure were estimated and compared between groups in the prematched and matched cohort using Kaplan-Meier analysis with a Log-rank test. AVVR ≥ moderate was estimated and compared between groups in the prematched and matched cohort using a cumulative incidence curve with Fine and Gray’s test (death or transplantation as a competing risk). The Cox proportional hazard model was used to identify risk factors associated with Fontan failure among the prematched cohort. Univariate analysis was first performed so that those with *p*-values less than 0.05 were further included in multivariate analysis. Dextrocardia, AVV operation before or at Fontan, and dominant RV, which were considered risk factors for Fontan outcomes, were also included in the multivariate analysis. Given few positive events in death or transplantation and AVVR ≥ moderate, multivariate analysis was not performed to identify associated risk factors.

## 3. Results

### 3.1 Patient Characteristics before and after PSM

The baseline characteristics are shown in the Table 1. Before PSM matching, the cohort comprised 69 patients in the RV-dominant group and 109 patients in the LV-dominant group. There was a significant difference in AVV morphology between the two groups (*p* < 0.001). Patients in the RV-dominant group were more likely to undergo fenestration during the Fontan and to have required an AVV operation before or after Fontan surgery (*p* < 0.05). Additionally, we observed a higher proportion of patients with combined atrial isomerism (*p* < 0.001) and anomalous pulmonary vein connection (*p <*0.05) in the prematched dominant RV group. After PSM, a total of 42 pairs were generated. All baseline characteristics were similar between the two groups (*p* > 0.05). The median age at Fontan operation was 6.0 in the matched RV-dominant group and 7.0 in the matched LV-dominant group.

**Table 1.** **Baseline characteristics before and after matching**.

		Before PSM				After PSM			
		Dominant RV	Dominant LV	SMD	*p*	Dominant RV	Dominant LV	SMD	*p*
		n = 69	n = 109			n = 42	n = 42		
Male		47 (68.1%)	70 (71.6%)	–0.081	0.594	27 (64.3%)	27 (64.3%)	<0.001	>0.999
Age at Fontan operation, y		5.0 (4.0–10.0)	5.0 (4.0–11.0)	–0.039	0.987	6.0 (3.0–12.3)	7.0 (4.0–13.0)	0.164	0.507
Weight at Fontan operation, kg		18.0 (14.5–24.5)	16.5 (14.0–28.0)	–0.080	0.494	18.0 (14.5–33.4)	18.3 (13.9–33.3)	–0.040	0.488
Fontan type					0.247				0.659
	LT	6 (8.7%)	4 (3.7%)	–0.267		5 (11.9%)	3 (7.1%)	–0.253	
	ECC	58 (84.1%)	92 (84.4%)	0.010		34 (81.0%)	37 (88.1%)	0.197	
	Others	5 (7.0%)	13 (11.0%)	0.144		3 (7.1%)	2 (4.8%)	–0.074	
Fontan fenestration		34 (49.3%)	36 (33.0%)	–0.346	0.031	18 (42.9%)	18 (42.9%)	<0.001	>0.999
AVV morphology					<0.001				0.931
	Mitral valve	6 (8.7%)	59 (54.1%)	0.912		6 (14.3%)	7 (16.7%)	0.048	
	Tricuspid valve	8 (11.6%)	1 (0.9%)	–1.120		1 (2.4%)	1 (2.4%)	<0.001	
	2 AVV	21 (30.4%)	31 (28.4%)	–0.044		20 (47.6%)	17 (40.5%)	–0.158	
	Common AVV	34 (49.3%)	18 (16.5%)	–0.882		15 (35.7%)	17 (40.5%)	0.128	
Atrial isomerism		23 (33.3%)	12 (11.0%)	–0.713	<0.001	9 (21.4%)	10 (23.8%)	0.076	0.794
Dextrocardia		6 (8.7%)	6 (5.5%)	–0.140	0.603	4 (9.5%)	5 (11.9%)	0.104	>0.999
Anomalous pulmonary vein connection		11 (15.9%)	7 (6.4%)	–0.388	0.040	5 (11.9%)	6 (14.3%)	0.097	0.746
AVVR ≥ moderate		15 (21.7%)	16 (14.7%)	–0.200	0.226	9 (21.4%)	9 (21.4%)	<0.001	>0.999
AVV operation before or at Fontan		18 (26.1%)	12 (11.0%)	–0.482	0.009	10 (23.8%)	6 (14.3%)	–0.204	0.266

PSM, propensity score matching; RV, right ventricle; LV, left ventricle; SMD, standardized mean difference; LT, lateral tunnel; ECC, extracardiac conduit; AVV, atrioventricular valve; AVVR, atrioventricular valve regurgitation.

### 3.2 Perioperative and Postoperative Outcomes

Before matching, chest drainage duration and length of postoperative hospitalization were significantly longer in the RV-dominant group than those in the LV-dominant group (*p* < 0.05), as shown in the Table 2. After matching, the cardiopulmonary bypass (CPB) time and aortic cross-clamping (ACC) time were similar between the two groups (*p* > 0.05). Similarly, no significant differences were noted in several critical postoperative parameters including mechanical ventilation time, length of intensive care unit (ICU) stay, chest drainage duration, length of postoperative hospitalization, in-hospital reintervention, and in-hospital mortality (*p* > 0.05). The mean follow-up periods were 8.0 ± 4.6 years in the matched RV-dominant group and 6.5 ± 4.7 years in the matched LV-dominant group (*p* > 0.05).

**Table 2.** **Perioperative and postoperative outcomes before and after matching**.

	Before PSM			After PSM		
	Dominant RV	Dominant LV	*p*	Dominant RV	Dominant LV	*p*
	n = 69	n = 109		n = 42	n = 42	
CPB time, min	142.0 (93.0–184.5)	118.0 (88.0–154.0)	0.084	125.0 (88.5–182.8)	126.0 (98.5–156.0)	0.989
ACC time, min	59.0 (0–90.5)	35.0 (0–78.0)	0.054	58.0 (0–89.3)	55.0 (0–83.0)	0.761
Mechanical ventilation time, h	8.7 (5.6–24.5)	8.7 (4.8–18.6)	0.532	8.2 (5.8–17.7)	11.2 (7.4–32.0)	0.207
Length of ICU stay, d	3.6 (1.7–5.9)	3.3 (1.6–5.8)	0.522	3.6 (1.7–6.1)	3.7 (1.6–6.7)	0.704
Chest drainage duration, d	13.0 (8.0–23.7)	10.0 (6.2–19.0)	0.049	14.5 (9.8–30.8)	13.3 (6.2–21.5)	0.248
Length of postoperative hospitalization	22.0 (15.0–34.0)	18.0 (12.0–25.0)	0.011	24.5 (15.0–37.0)	22.0 (14.0–28.0)	0.248
In-hospital reintervention	6 (8.7%)	11 (10.1%)	0.758	3 (7.1%)	5 (11.9%)	0.710
In-hospital mortality	3 (4.3%)	6 (5.5%)	>0.999	1 (2.4%)	3 (7.1%)	0.608
Period of follow-up, y	7.9 ± 4.4	7.4 ± 4.6	0.451	8.0 ± 4.6	6.5 ± 4.7	0.163

PSM, propensity score matching; RV, right ventricle; LV, left ventricle; CPB, cardiopulmonary bypass; ACC, aortic cross-clamping; ICU, intensive care unit.

### 3.3 Long-Term Outcomes of RV and LV Dominance

**Fig. 2.**
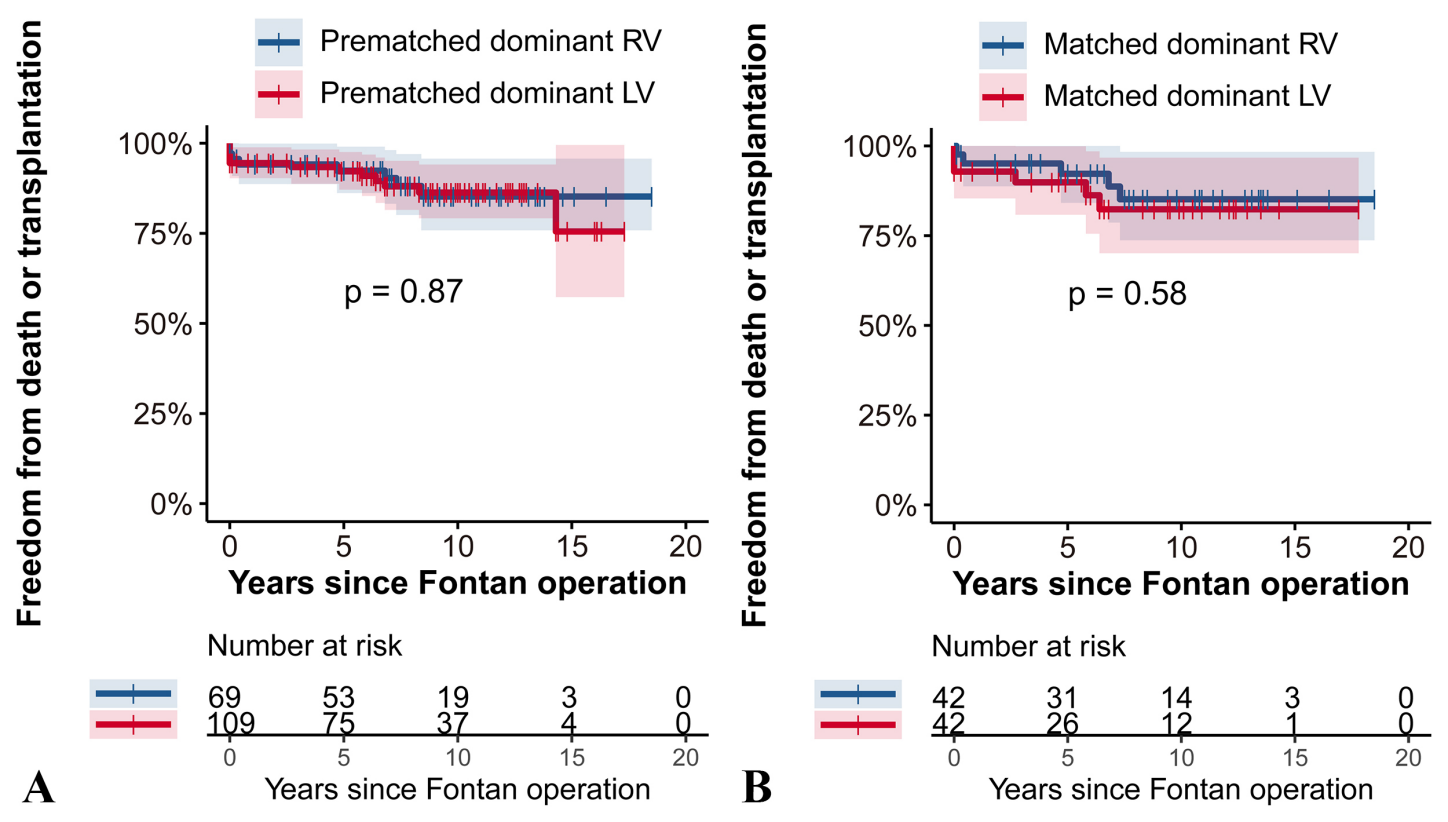
**Freedom from death or transplantation RV vs. LV dominance analyzed with the Log-rank test in the prematched cohort (A) and the matched cohort (B).** This figure illustrates the comparison of survival without death or transplantation between patients with dominant RV and LV before and after propensity score matching. (A) In the prematched cohort there were no significant differences in freedom from death or transplantation between the RV and LV groups (*p* = 0.87). (B) Similarly, in the matched cohort (B), survival rates remained comparable with no significant difference detected (*p* = 0.58). RV, right ventricle; LV, left ventricle.

As shown in the Fig. 2, there were no significant differences in freedom from death or transplantation between the two groups in either the prematched or matched cohorts (*p* > 0.05). Before matching, the 10-year freedom from death or transplantation was estimated to be 85% ± 5% in the RV-dominant group, and 81% ± 6% in the LV-dominant group (Fig. 2A). After matching, these rates were similar at 84% ± 7% for RV and 81% ± 7% for LV (Fig. 2B).

**Fig. 3.**
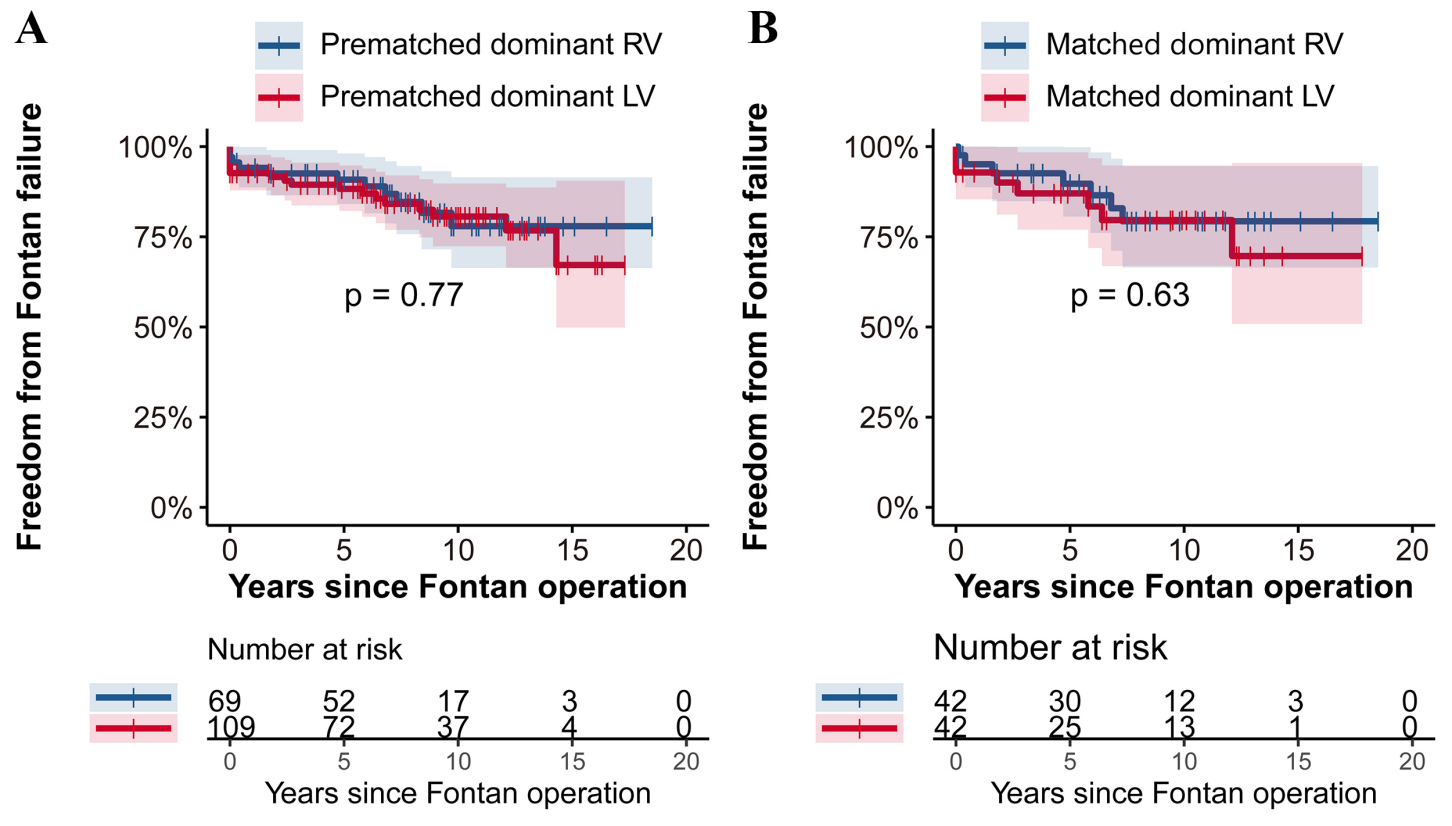
**Freedom from Fontan failure: RV vs. LV group with Log-rank test in the prematched cohort (A) and the matched cohort (B). **This figure presents the comparison of freedom from Fontan failure between patients with dominant RV and LV before and after propensity score matching. (A) In the prematched cohort, the freedom from Fontan failure was comparably similar between the RV and LV groups (*p* = 0.77). (B) The trend continued in the matched cohort (B), where no significant difference in freedom from Fontan failure was observed between the two groups (*p* = 0.63). RV, right ventricle; LV, left ventricle.

Fig. 3 further illustrates the similarity in outcomes concerning freedom from Fontan failure between the groups. Pre- and post-matching analyses revealed no significant differences (*p* > 0.05). The prematched RV group showed a 10-year freedom from Fontan failure rate of 78% ± 6%, closely matching the LV group, with 76% ± 6% (Fig. 3A). Following matching, both groups demonstrated comparable rates, with 78% ± 8% in the matched dominant RV group and 75% ± 8% in the matched dominant LV group (Fig. 3B). These results highlight consistent long-term outcomes across both ventricular dominance groups, indicating that ventricular dominance may not significantly influence survival or freedom from Fontan failure rates.

**Fig. 4.**
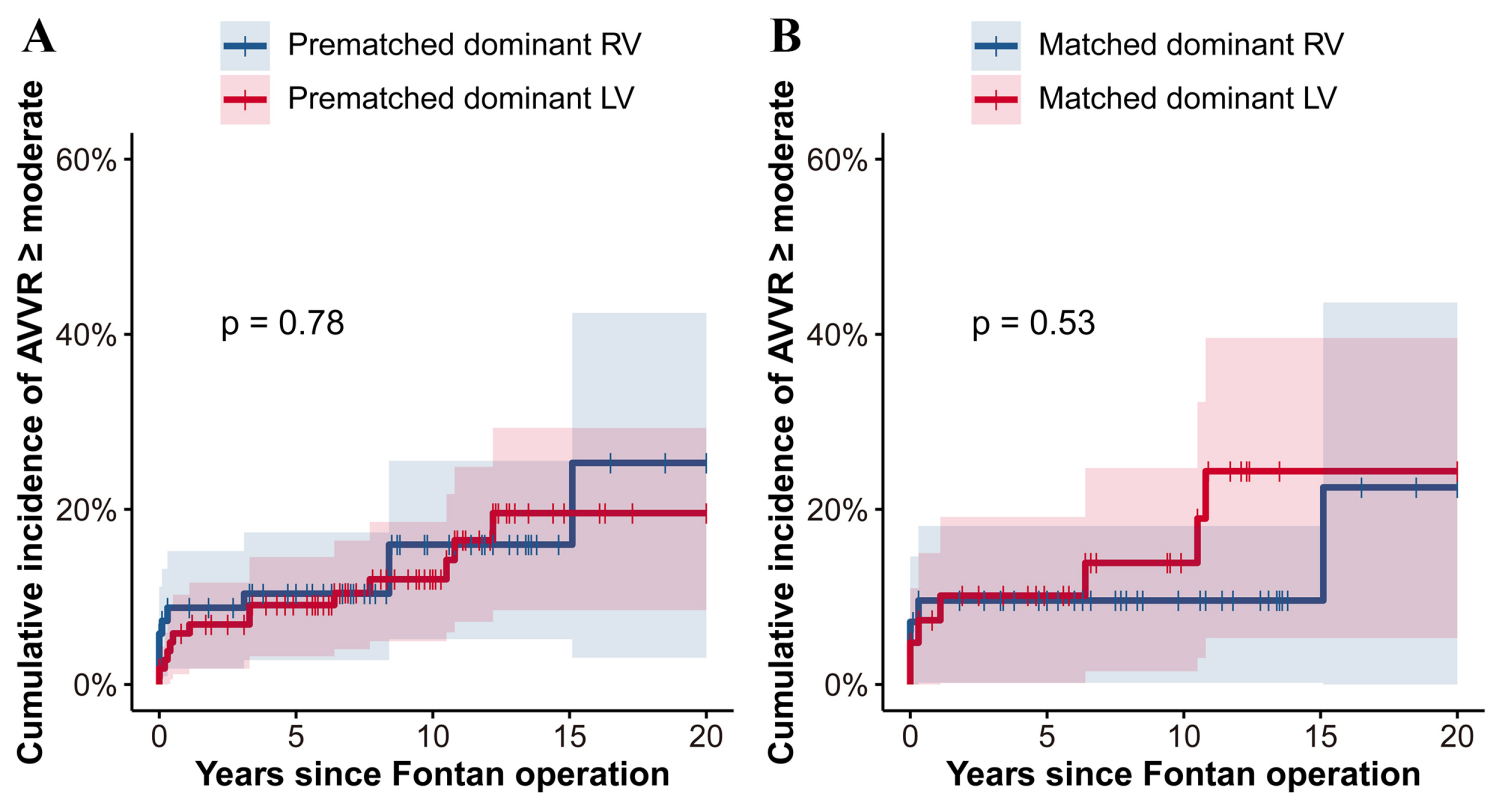
**Cumulative incidence of AVVR ≥ moderate: RV vs. LV dominance compared using Gray’s test in the prematched cohort (A) and the matched cohort (B).** This figure evaluates the cumulative incidence of moderate or greater AVVR in patients with dominant RV and LV before and after propensity score matching. (A) In the prematched cohort Fontan patients with RV-dominant morphology showed a cumulative incidence of AVVR ≥ moderate comparable to those with LV-dominant morphology (*p* = 0.78). (B) Similarly, in the matched cohort no significant difference in the cumulative incidence of AVVR ≥ moderate was observed between the two groups (*p* = 0.53). AVVR, atrioventricular valve regurgitation; RV, right ventricle; LV, left ventricle.

Fig. 4 illustrates the cumulative incidence of of moderate or greater AVVR ≥ in both prematched and matched cohorts, comparing dominant RV and LV groups. Analysis showed no significant difference between groups in either cohort (*p* > 0.05). In the prematched RV-dominant group, the estimated 10-year cumulative incidence of AVVR ≥ moderate was 16% ± 5% compared with 21% ± 6% in the prematched LV-dominant group (Fig. 4A). In the matched cohort, the estimated cumulative incidence of AVVR ≥ moderate at 10 years after Fontan operation was 11% ± 5% in the RV-dominant group, in contrast to 15% ± 6% in the LV-dominant group (Fig. 4B).

### 3.4 Multivariate Analysis for Fontan Failure

Table 3 outlines the results from univariate and multivariate Cox proportional hazard analyses identifying predictors of Fontan failure. Significant factors associated with an increased risk of Fontan failure in the univariate analysis included atrial isomerism, anomalous pulmonary vein connection, CPB time, mechanical ventilation time, and length of ICU stay (*p* < 0.05). However, when these variables were evaluated in a multivariate analysis, only atrial isomerism (hazard ratio [HR] = 2.909, 95% confidence interval [CI] = 1.176–7.196), and mechanical ventilation time (HR = 1.010, 95% CI = 1.003–1.018) remained statistically significant predictors of Fontan failure (*p* < 0.05). Crucially, RV dominance was not identified as a risk factor for Fontan failure in either univariate or multivariate analysis (*p* > 0.05) suggesting that the presence of an RV-dominant configuration does not independently predict the outcome of Fontan failure.

**Table 3.** **Univariate and multivariate analysis for Fontan failure**.

	Univariate		Multivariate	
	HR (95% CI)	*p*	HR (95% CI)	*p*
Dominant RV	0.894 (0.425–1.881)	0.768	0.559 (0.249–1.257)	0.159
Atrial isomerism	3.677 (1.779–7.597)	<0.001	2.909 (1.176–7.196)	0.021
Dextrocardia	1.362 (0.413–4.491)	0.612	1.373 (0.385–4.896)	0.625
Anomalous pulmonary vein connection	2.693 (1.096–6.621)	0.031	1.432 (0.413–4.962)	0.571
AVV operation before or at Fontan	1.650 (0.703–3.872)	0.250	1.070 (0.402–2.852)	0.892
CPB time, min	1.006 (1.002–1.010)	0.001	1.001 (0.997–1.005)	0.490
Mechanical ventilation time, h	1.004 (1.002–1.006)	<0.001	1.010 (1.003–1.018)	0.007
Length of ICU stay, d	1.049 (1.008–1.091)	0.018	0.860 (0.729–1.016)	0.075

HR, hazard ratio; CI, confidence interval; RV, right ventricle; AVV, atrioventricular valve; CPB, cardiopulmonary bypass; ICU, intensive care unit.

## 4. Discussion

The association between RV-dominance and poorer long-term outcomes in Fontan circulation is a subject of ongoing debate. Our study employed PSM to balance patient characteristics between pre- and intra-Fontan status, focusing on those with non-HLHS, to compare outcomes between RV and LV dominance. Our findings revealed that: (1) The cumulative incidence of AVVR was similar between the two groups in both the prematched cohort and the matched cohort. (2) Dominant ventricular morphology did not significantly impact the likelihood of long-term freedom from death or transplantation and Fontan failure in either the prematched or matched cohorts. These results suggest that the ventricular morphology, whether RV or LV dominance, does not decisively influence the long-term success of Fontan circulation in the non-HLHS patient population.

Anatomical and embryological distinctions between the RV and LV, along with differences in AVV, suggest the RV might be more susceptible to pressure-overload when it assumes responsibility for systemic circulation, potentially, leading to or accelerating the occurrence and progression of AVVR [16]. Indeed, previous studies [17] revealed a higher rate of AAVR deterioration and progression AVVR in patients with RV dominance compared to those with LV dominance in Fontan circulation. Of note, instances of moderate or greater AVVR at the time of Fontan operations and subsequent AVV repair or replacement were more common in the dominant RV group. However, specific details regarding AVV morphology in these comparisons remained unclear.

Contrary to these observations, our study found no significant difference in the cumulative incidence of moderate or greater AVVR between the prematched RV and LV dominant groups. Further, after matching patients for similar clinical AVV status, we observed equivalent rates of AVVR across RV and LV dominant Fontan patients. This echoes findings from a recent retrospective study of 174 Fontan patients with atrioventricular septal defect, showing no significant difference of moderate or greater AVVR between the two groups [18]. This suggests that AVV morphology and anatomy may play a more critical role in AVVR development than ventricular dominance itself.

Moreover, in our matched cohorts, the 10-year cumulative incidence of moderate or greater AVVR was closely matched between the RV and LV dominant groups, at 11% ± 5% and 15% ± 6%, respectively, which was consistent with previous studies [19]. For instance, King *et al*. [17] reported a 10% incidence of moderate or greater AVVR 10 years after Fontan surgery in a large retrospective study involving 1703 patients. These outcomes collectively indicate that while ventricular dominance may not significantly influence AVVR progression, the specific morphological and anatomical features of the AVV are crucial determinants of AVVR development in Fontan patients.

Our study demonstrates that the long-term outcomes following Fontan operation, specifically freedom from death or transplantation and freedom from Fontan failure, do not significantly differ between patients who are RV-dominant and LV-dominant. In the matched groups, the 10-year freedom from death or transplantation was 84% ± 7% in the matched RV-dominant group, similar to 81% ± 7% in the matched LV-dominant group (*p* > 0.05). Additionally, the 10-year freedom from Fontan failure was 78% ± 8% in the matched RV-dominant group versus 75% ± 8% in the matched LV-dominant group (*p* > 0.05). Furthermore, RV dominance was not identified as an independent risk factor for Fontan failure in our multivariate analysis.

This finding aligns with previous studies that presents varied and often contradictory evidence on the impact of dominant ventricular morphology on long-term outcomes in Fontan circulation [11, 20, 21, 22, 23, 24, 25]. For example, Hosein *et al*. [26] retrospectively analyzed 406 Fontan patients (60% HLHS in the RV-dominant group) during the mean follow-up of 6.1 years, showing that ventricular morphology did not adversely influence short-term or long-term outcomes of Fontan patients. The freedom from death or transplantation at 5 years and 10 years after the Fontan operation was 90% and 86%, respectively, which was consistent with our findings [26]. These consistencies across studies suggest that despite the theoretical implications of ventricular morphology on post-Fontan prognosis, the actual influence may be minimal, underscoring the need for a nuanced understanding of individual patient characteristics and the multifactorial nature of outcomes following Fontan surgery.

In contrast, Moon *et al*. [11] conducted a retrospective study of 1162 patients with a direct focus on ventricular morphology, with 71% of these patients classified as RV-dominant with HLHS. With a mean follow-up of 8.3 years, they concluded that RV dominance impaired long-term outcomes when compared to the LV-dominant group, potentially due to AVVR deterioration and impaired ventricle function [11]. Notably, they reported a 10-year transplantation-free survival rate of 90% in the RV-dominant group, significantly lower than 92% in the LV-dominant group (*p* < 0.05), which differed from our findings [11].

Several factors may account for these contrasting results. First, the inherent heterogeneity among Fontan patients, who present with a wide variety of congenital cardiac structural abnormalities, may contribute to these discrepancies. Indeed, many previous studies showed an imbalance in baseline anatomic characteristics between the two groups [11, 18]. Our application of PSM aimed to reduce the selection bias, resulting in outcomes that were similar between groups. This suggests the possibility that inherent cardiac structural abnormalities, rather than RV-dominant morphology influence long-term outcomes. Secondly, the incidence of moderate or greater AVVR, a factor known to affect outcomes due to its contribution to volume overloading, ventricular dilation, and increased central venous pressure, was maintained between groups in our study [5]. This similarity in AVVR progression supports the idea that ventricular dominance may not be the primary determinant of outcomes. Thirdly, follow-up durations in our study were slightly shorter than in Moon’s study, with 8.0 years for RV dominance and 6.5 years for LV dominance in our study vs. 8.3 years in Moon’s study [11]. It’s possible that the systemic circulation supported by the dominant RV could remain in a compensatory state within this timeframe, leading to outcomes that did not significantly differ from those of the LV-dominant group. Lastly, our exclusion of HLHS patients, who are generally at higher risk for adverse outcomes, may have minimized the perceived disadvantages of the RV-dominant group, contributing to our findings of similar outcomes [12]. The exclusion criteria perhaps weakened the disadvantage of the RV-dominant group, partially contributing to the finial similar results. Regardless, despite differing perspectives in the literature, our research suggests that the long-term prognosis for dominant RV and LV in non-HLHS Fontan patients may be similar, at least within the duration of our follow-up period.

There were several limitations in our study. First, this is a single-center study with a retrospective design, which may limit the applicability of the findings to broader populations. Secondly, the relatively small sample size of Fontan patients both before and after PSM could diminish the statistical power of our results. Finally, although cardiac magnetic resonance imaging remains the gold standard for determining functional ventricular dominance through quantification of cardiac output and stroke volume, it was not typically utilized before Fontan operations in our hospital due to the high cost and long waiting periods for appointments. Consequently, ventricular dominance was assessed using echocardiography, a method that may not provide an ideal assessment for all patients. This potentially led to the exclusion of some patients categorized as having indeterminate or biventricular morphology from this analysis.

## 5. Conclusions

The RV dominance does not seem to adversely influence the long-term outcomes in non-HLHS Fontan circulation. The likelihood of experiencing moderate or greater AVVR, transplantation-free survival, and avoiding Fontan failure were similar between RV and LV dominant groups within in the same pre- and intra-Fontan conditions.

## Data Availability

Data are available from the corresponding author upon reasonable request.

## Abbreviations

SV, single ventricle; RV, right ventricle; LV, left ventricle; HLHS, hypoplastic left heart syndrome; PSM, propensity score matching; AVVR, atrioventricular valve regurgitation; AVV, atrioventricular valve.
